# Digitized subwavelength surface structure on silicon platform for wavelength-/polarization-/charge-diverse optical vortex generation

**DOI:** 10.1515/nanoph-2022-0395

**Published:** 2022-09-19

**Authors:** Xiaoping Cao, Nan Zhou, Shuang Zheng, Shengqian Gao, Yuntao Zhu, Mingbo He, Xinlun Cai, Jian Wang

**Affiliations:** Wuhan National Laboratory for Optoelectronics and School of Optical and Electronic Information, Huazhong University of Science and Technology, Wuhan 430074, Hubei, China; Optics Valley Laboratory, Wuhan 430074, Hubei, China; State Key Laboratory of Optoelectronic Materials and Technologies and School of Physics and Engineering, Sun Yatsen University, Guangzhou 510275, China

**Keywords:** integrated optics devices, optical communications, optical vortices, subwavelength structures

## Abstract

Optical vortices carrying orbital angular momentum (OAM) have recently attracted increasing interest for providing an additional degree of freedom for capacity scaling in optical communications. The optical vortex generator is an essential component to facilitate OAM-enabled optical communications. Traditional devices face challenges of limited compactness, narrow bandwidth, and first-order OAM modes. Here, using the direct-binary search (DBS) optimization algorithm, we design, fabricate, and demonstrate a digitized subwavelength surface structure on silicon platform for the generation of wavelength-/polarization-/charge-diverse optical vortices. It features an ultra-compact footprint (∼3.6 × 3.6 μm^2^) and ultra-wide bandwidth (1480–1630 nm), supporting two polarizations (*x*-pol., *y*-pol.) and high-order OAM modes (OAM_+1_, OAM_−1_, OAM_+2_, OAM_−2_) with high purity of larger than 84%. The mode crosstalk matrix is measured in the experiment with favorable performance. When generating *x*-pol. OAM_+1_, *x*-pol. OAM_−1_, *y*-pol. OAM_+1_, and y-pol. OAM_−1_ mode, the crosstalk of the worst case is less than −14 dB. When generating OAM_+1_, OAM_−1_, OAM_+2_, and OAM_−2_ mode, the crosstalk between any two OAM modes is less than −10 dB, and the lowest crosstalk is about −17 dB. In addition, we also show the possibility for generating much higher-order OAM modes (e.g. OAM_+3_, OAM_−3_, OAM_+4_, and OAM_−4_) with the digitized subwavelength surface structure. The wavelength-/polarization-/charge-diverse optical vortex generator enables the full access of multiple physical dimensions (wavelength, polarization, space) of lightwaves. The demonstrations may open up new perspectives for chip-scale solutions to multi-dimensional multiplexing optical communications.

## 1 Introduction

It is well known that light traveling through space may rotate, carrying angular momentum. Aside from the circular polarization rotation referring to the spin angular momentum (SAM), the light can also rotate with its spatial phase structure, leading to a specified helical phase front and carrying additional angular momentum, namely the orbital angular momentum (OAM) [1]. Generally, a light beam carrying OAM, equivalent to a value of *lћ* per photon (*l*: topological charge), is called optical vortex with an annular intensity structure incorporated with an azimuthal phase structure of exp(i*lϕ*), where *ϕ* is the azimuthal angle. The topological charge or mode order *l*, in principle, can take any integer value, positive or negative. Such distinct features of optical vortex have fueled multitudinous researches and applications in many branches of optical physics, including optical manipulation, optical tweezers, optical spanners, particle trapping, memory, microscopy, imaging, sensing, metrology, astronomy, nonlinear interaction, and quantum science [2–14]. Very recently, OAM-carrying optical vortex has also attracted increasing interest in optical communications [15–20]. Remarkably, being fully compatible with existing multiplexing resources such as time, wavelength, and polarization in optical communications, OAM as a subset of spatial modes provides an additional physical dimension for multiplexing, which allows the continuous scaling of transmission capacity and spectral efficiency. OAM beams have helical phase fronts with the number of intertwined helices and the direction depending on the magnitude and the sign of *l*, respectively. The topological charge *l*, also referring to the mode order, can take an unbounded value theoretically, so an infinite number of OAM beams could be accommodated provided an infinitely large aperture. Even for a finite aperture in a practical communication system, multiple OAM beams can still be used to assure the capacity scaling by OAM multiplexing. The space-division multiplexing (SDM) exploiting OAM mode basis provides a promising approach to achieve large capacity beyond traditional multiplexing techniques for free-space, underwater, and fiber optical communications [15–40].

The generation of OAM-carrying optical vortices is essential for almost all the OAM-enabled applications, including OAM-based optical communications. Unlike ordinary light beams with a planar phasefront, the generation of optical vortices characterized by doughnut intensity profiles and helical phasefronts usually relies on bulky devices used for mode conversion, such as cylindrical lens pairs, spiral phase plates, diffractive optical elements, and spatial light modulators [41–44]. Additionally, resonator cavities, q-plates, metamaterials, and metasurfaces are also used for generating optical vortices [45–55]. Very recently, for the sake of miniaturization and integration, photonic integrated devices have been demonstrated to generate optical vortices [56–68]. In particular, silicon photonic integration platform plays an increasingly important role because of its miniaturization, reliability, scalability, and complementary metal-oxide semiconductor (CMOS) compatibility for low-cost mass production [69, 70]. The previous works based on silicon photonic platform such as waveguide phase arrays, whispering-gallery-mode resonators, and holographic gratings succeeded in generating optical vortices with impressive performance. However, challenges remain especially in terms of limited compactness, narrow bandwidth, and first-order OAM modes only. Remarkably, the full compatibility of OAM multiplexing with existing well-established multiplexing techniques such as wavelength-division multiplexing (WDM) and polarization-division multiplexing (PDM) is essential to OAM-assisted SDM optical communications. In this scenario, ultra-compact nanophotonic devices based on silicon platform for wavelength-/polarization-/charge-diverse optical vortex generation are of great research value. The wavelength-/polarization-/charge-diversity can facilitate OAM multiplexing with high-order optical vortices and its compatibility with WDM (ultra-broadband property) and PDM techniques.

In this paper, based on a nonlinear search algorithm called direct-binary search (DBS), we design, fabricate, and demonstrate a digitized subwavelength surface structure on silicon platform for the generation of wavelength-/polarization-/charge-diverse optical vortices with outstanding performance. Such nanostructured silicon photonic device enables the generation of optical vortices with two OAM values (OAM_+1_, OAM_−1_) and two polarization states (*x*-/*y*-polarization) in a wide wavelength range from 1480 to 1630 nm. Moreover, diverse optical vortex generation beyond the first-order OAM is also available, e.g. ultra-broadband and polarization-diversity generation of optical vortices with different OAM values (OAM_+1_, OAM_−1_, OAM_+2_, OAM_−2_) is demonstrated here. The mode properties are characterized in detail in the experiment, as well as the mode crosstalk.

## 2 Methods

**Figure 1:**
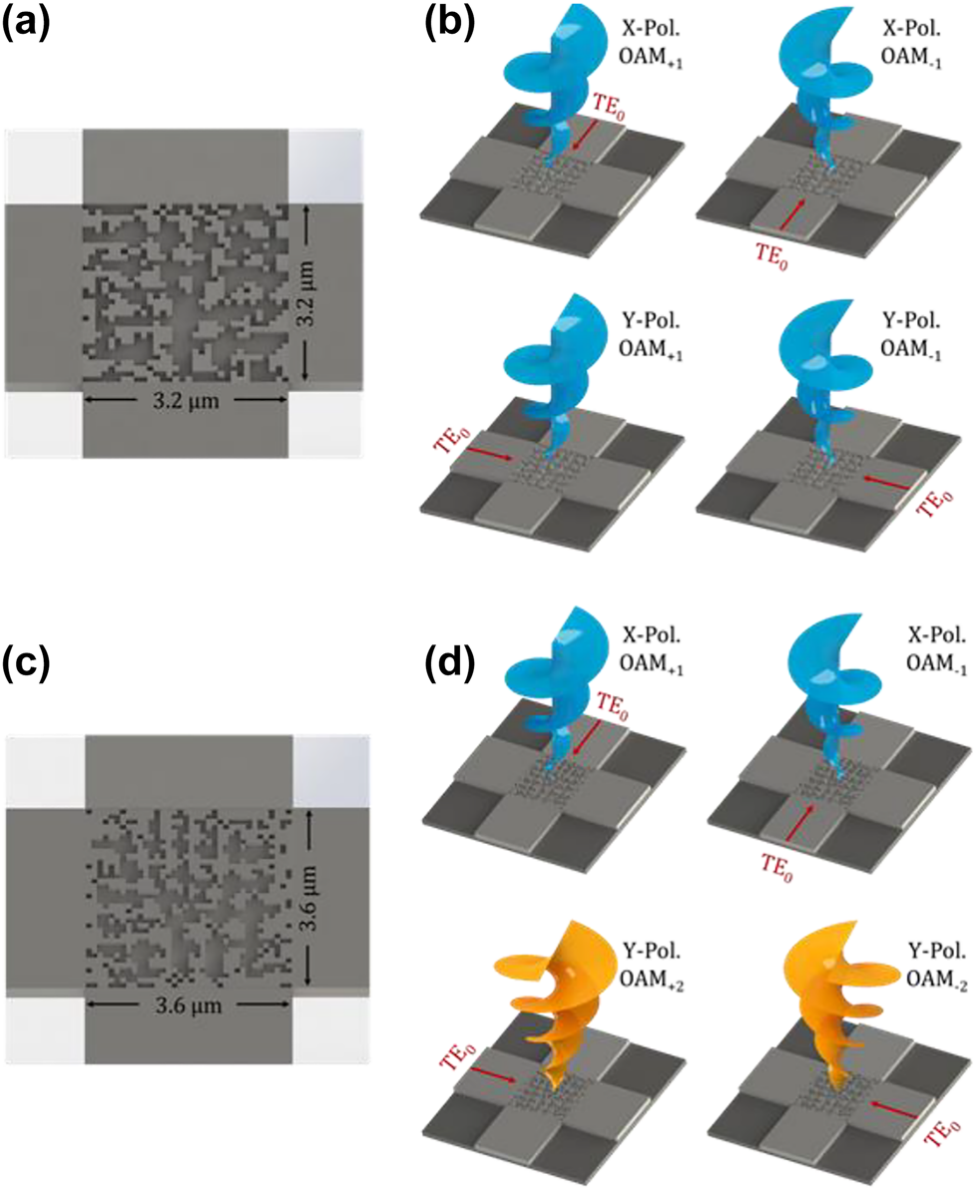
Schematic illustration of the ultra-compact wavelength-/polarization-/charge-diverse optical vortex generators. (a) and (b) The generator 1 with optimized digitized nanostructure for generating *x*-pol. OAM_+1_, *x*-pol. OAM_−1_, *y*-pol. OAM_+1_, and *y*-pol. OAM_−1_ mode under different incident conditions. (c) and (d) The generator 2 with optimized digitized nanostructure for generating *x*-pol. OAM_+1_, *x*-pol. OAM_−1_, *y*-pol. OAM_+2_, and *y*-pol. OAM_−2_ mode under different incident conditions.

Firstly, we design a chip-scale optical vortex generator used for the generation of *x*-pol. OAM_+1_, *x*-pol. OAM_−1_, *y*-pol. OAM_+1_, and *y*-pol. OAM_−1_ mode. For the generator 1 illustrated in Figure 1(a) and (b), the fundamental TE_0_ mode respectively from four input ports in different directions is coupled into the vertically emitted optical vortices with specific topological charge value and polarization state after propagation through the 2D digitized subwavelength surface structure on silicon platform. It should be noted that the optical vortex is mainly characterized by the 3D spiral phase structure, thus the generation of optical vortices primarily depends on the phase modulation of the incident in-plane guided mode (fundamental TE_0_ mode), which could be realized by a subwavelength surface structure such as holographic fork grating. Based on this method depending on the modulation of the propagation phase delay, the first-order OAM mode can be generated with high purity. However, significant challenge remains to further improve the quality of the generated optical vortices and generate high-order optical vortices, where more accurate phase modulation during the conversion from in-plane guided mode to free-space optical vortex is highly desired. Here, localized phase modulation is introduced by further digitizing the conventional holographic fork grating into a more general subwavelength surface structure. Shown in Figure 1(a) is the zoom-in details of the proposed digitized subwavelength surface structure of the optical vortex generator, which is designed and fabricated on a standard silicon-on-insulator (SOI) platform with a normal 220-nm silicon layer and 2-μm buried oxide layer. The designed digitized nanostructure region is shallow etched down normally 60 nm with a compact footprint of 3.2 × 3.2 μm^2^, discretized by numerous 100 nm × 100 nm silicon/silicon dioxide pillars, namely “pixels.” Thus, we call it digitized subwavelength surface structure on silicon platform.

This type of digitized nanophotonic structure has been increasing popular to design photonic integrated devices with arbitrary topologies used for multiple novel functionalities [71–79]. Unlike conventional design techniques with only a small amount of parameters optimized one by one, the digitized design based on optimization algorithms allows the full parameter space searching of possible structures even without user intervention, which could be seen as a promising technical innovation. Recently, a crowd of powerful optimization strategies have been developed for nanophotonic design, ranging from heuristic optimization methods to topological design methods. Several classical heuristic optimization methods, such as genetic algorithm, simulated annealing algorithm, particle swarm algorithm, and attractor selection algorithm, have traditionally been applied to many fields. Critically, most of them are built on a somewhat limited parameterization of the solution space and require abundant plentiful random testing of different parameter sets, putting the applications in relatively complicated models under high computational cost. To avoid the test of a large number of possible solutions before finding a satisfactory one, an inverse design method is introduced under the inspiration of the variable density method used in continuum topology optimization [75–79]. The device structure could be discretely parametrized into a 2D image with each pixel corresponding to the value of the permittivity. In general, the optimization procedure could be decomposed into two stages. Firstly, the permittivity of each pixel is allowed to vary continuously between that of silicon and silicon oxide, where the gradient descent method is normally used to generate a well-preformed initial structure. Then a binary discrete constraint is imposed on the permittivity of them because the fabrication material is only chosen to be silicon or silicon oxide.

Although many basic integrated devices have been designed with remarkable performance and compact footprint in this way, the drawback is reasonably obvious as well. Above all, a good initial condition is of crucial importance to the entire optimization design process. As mentioned, the continuous optimization stage is essentially relied on the descent gradient calculated with precise full wave electromagnetic simulation, which will still consume a lot of computing resources even with the adjoint method [80, 81]. Besides, the continuous parameter optimization is normally ended with weakly modulated permittivity between silicon and silicon oxide, leading to severe degradation of device performance after determining the final binary discrete structure, and this problem could not be solved in essence with the recently proposed simple approximation or the level set method [82]. In this work, we get around this problem by defining the material of each pixel to be digitized binary at first, and the size is chosen to be 100 nm × 100 nm, totally satisfying requirements of the manufacturing process. Then the design is followed by the DBS optimization algorithm. Originally the initial structure is generated randomly by MATLAB, but the optimization turns out to be sensitive to the starting designs, making it hardly to get an optimal design. Then comes the question how to generate a well-performed initial structure without the continuous stage. As stated above, modulation of the propagation phase could be realized by a surface holographic fork grating [67]. Hence, here an initial structure of holographic fork grating is chosen based on our previous work, which could be used to generate polarization diversity first-order OAM modes. Next, the pixels are chosen to switch its state in a random order, and a figure-of-merit (FOM) is calculated each time for comparison. The FOM is defined as the average phase purity calculated for all emitted OAM modes coupled from different input ports of the device. The pixel state is retained once the FOM is improved, if not, the state is reversed and the algorithm proceeds to the next pixel. One iteration could iterate over all the pixels and more iterations will be executed until no more improvement exhibits on the FOM. In the end, the final structure is numerically simulated with three-dimensional finite-difference time-domain (3D-FDTD) method to prove the validity of the designed digitized subwavelength surface structure on silicon platform for wavelength-/polarization-/charge-diverse optical vortex generation.

**Figure 2:**
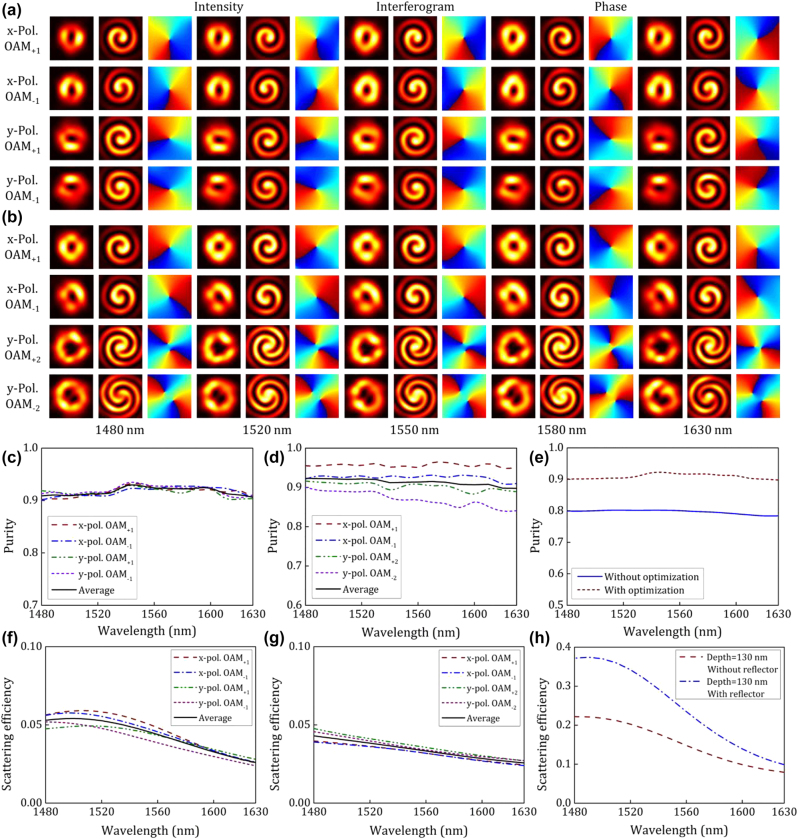
Simulation results for characterizing the wavelength-/polarization-/charge-diverse optical vortex generator. (a) Intensity profiles, interferograms, and phase distributions of the generated OAM modes from generator 1 from 1480 to 1630 nm, respectively. (b) Intensity profiles, interferograms, and phase distributions of the generated OAM modes from generator 2 from 1480 to 1630 nm, respectively. (c) Calculated purity of the generated OAM modes from generator 1 versus wavelength. (d) Calculated purity of the generated OAM modes from generator 2 versus wavelength. (e) Comparison of the average mode purity of the generated OAM modes from generator 1 between the initial discrete structure and the nanostructure after DBS optimization. (f) Calculated scattering efficiency of the generated OAM modes from generator 1 versus wavelength. (g) Calculated scattering efficiency of the generated OAM modes from generator 2 versus wavelength. (h) Comparison of the scattering efficiency of the generated *y*-pol. OAM_+1_ mode between the designed OAM generator with and without a reflection layer under the etching depth of 130 nm.

In Figure 2(a), we give the detailed simulation results of the generated wavelength-/polarization-/charge-diverse OAM modes with two mode orders and two polarization states ranging from 1480 to 1630 nm, respectively. The intensity profiles of the generated OAM modes is firstly studied with an annular structure, whose far field is calculated by the scalar diffraction theory. To evaluate the quality of the generated OAM modes more accurately, we further study the mode purity of them, so that their interferograms and phase distributions are given as well. The interferogram is obtained via the interference between the generated OAM mode and a coherent reference Gaussian beam, in which the number and hand of spirals correspond to the order information including the magnitude and sign of the OAM mode. Besides, one can tell the mode order and evaluate the phase purity from the extracted phase distribution. Remarkably, based on the synthesis of a series of digitized subwavelength nanostructures with low *Q* resonance, the designed OAM generator exhibits well-deserved broadband characteristic. Hence, the property characterization of the generated OAM modes turns out to be basically identical in the simulation in the C-band. Even within a wider wavelength range, little degradation happens to the calculated purity of the generated OAM modes. As shown in Figure 2(c), all the four different OAM modes are generated with high purity of larger than 0.88 when varying the wavelength from 1480 to 1630 nm. Considering that four OAM modes vary slightly from each other and share the same tendency of wavelength-dependent purity, one can see that the average purity reaches a maximum value of 0.93 around 1550 nm, which is designed to be the central wavelength. We compare the performance between the nanostructure after DBS optimization and the initial discrete structure of holographic fork grating. Taking a glance at the average purity value of the generated OAM modes in Figure 2(e), one can find out an obvious improvement above 10% in the mode purity using the nanostructure after DBS optimization. It should be noted that the initial structure prepared for iterative optimization is discretized by digitized binary from the ideal holographic fork grating structure. The performance degradation would be inevitably introduced in this discrete process, but it is necessary to introduce the localized phase control. Followed by the DBS iterative optimization, the OAM modes could be generated with even better quality assisted by the more precise phase control.

In order to fully exploit the spatial dimension of lightwaves in OAM-assisted SDM optical communications, high-order OAM modes beyond OAM_+1_ and OAM_−1_ mode are also expected to be emitted from an OAM generator with such optimized digitized nanostructure. Based on the same DBS iterative optimization method, we further study a charge diversity optical vortex generator supporting high-order OAM modes. For experimental demonstration of high-order OAM generator, the generator 2 is designed to support OAM_+1_, OAM_−1_, OAM_+2_, and OAM_−2_ mode with light incident from four input ports, respectively, and the schematic illustration is shown in Figure 1(b). Following the same design principle, what differs from the generator 1 is that the designed nanostructure region contains 36 × 36 pixels, which is shallowly etched down 60 nm with each pixel size of 100 nm × 100 nm. The number of pixels is expanded slightly, which could give rise to a relatively more refined phase control to generate high-order OAM modes.

From the simulation results shown in Figure 2(b), one can clearly identify the characteristics of the generated four charge diversity OAM modes (OAM_+1_, OAM_−1_, OAM_+2_, OAM_−2_). Slight nonuniform distribution is observed in the far-field intensity profile, which could be ascribed to the inhomogeneous distribution of digitized nanostructured pixels, each differing in emission efficiency when the in-plane guided mode propagates through the nanostructured region. Instead of the helical phase distribution, more extra factors on the intensity distribution could be considered in the global optimization to avoid this fact. From the simulated interferograms and phase distributions, one can easily tell that the OAM modes not only from the number and direction of the spirals but also from the phase change (−2*π*, 2*π*, −4*π*, 4*π*) along the azimuthal direction. The calculated mode purity exhibits slight difference between the first-order OAM modes and the second-order OAM modes, as shown in Figure 2(d). Although it is reasonable that the purity of OAM_±2_ modes is slightly lower than the OAM_±1_ modes, OAM modes with the same |*l*| but opposite sign also slightly differ in purity. This could be ascribed to the fact that the judgment condition of FOM only considers the average purity of all generated OAM modes. Since the generation of high-purity OAM_±2_ modes is much harder than OAM_±1_ modes, iterative optimization under such FOM might sacrifice the purity of OAM_±2_ modes and improve OAM_±1_ modes so as to achieve a greater FOM. That is why the mode purity of OAM_±1_ modes for generator 2 is even high than generator 1, while the mode purity of OAM_±2_ modes is not that satisfying. To avoid this problem, increasing the proportion of the mode purity of high-order OAM modes would be an efficient method. Simulation results for generator 2 show that the maximum purity of the OAM_+1_ mode is 0.96 while it is 0.93 for the OAM_−1_ mode. The purity of the OAM_+2_ mode could reach 0.92 while OAM_−2_ mode 0.9. The purity of the OAM_+1_ mode and OAM_−1_ mode is larger than 0.94 and 0.91, respectively, within the wavelength range from 1480 to 1630 nm. On the other side, the purity of the OAM_+2_ mode and OAM_−2_ mode slightly decreases from 0.89 to 0.84 with the increase of the wavelength from 1480 to 1630 nm, indicating a slight performance degradation in terms of bandwidth.

Besides, the scattering efficiency of the proposed optical vortex generators is studied as well, and the simulation results are exhibited in Figure 2(f) and (g). The scattering efficiency decreases with increasing working wavelength. For the fabricated generator 1, the average scattering efficiency is about 5% at the wavelength of 1550 nm and about 4% for the fabricated generator 2. It could be noticed that the conversion efficiency is relatively low, which could be attributed to the following reasons. Firstly, it is well known that the scattering efficiency of such diffraction structures is largely proportional to the structure size. Considering here we demonstrate a digitized subwavelength surface structure on silicon integrated platform for optical vortex generation, which features an ultra-compact footprint of no larger than 3.6 × 3.6 μm^2^, the scattering efficiency is limited undoubtedly. Secondly, the etching depth of the subwavelength surface structure determines the scattering efficiency to a large extend. Although only the average purity of the generated modes is considered in the optimization process, relative simulation has also been taken effort by adding the scattering efficiency into the judgment condition of FOM. It turns out that the mode purity would usually be improved at the expense of scattering efficiency in the optimization process when the FOM is set to be the sum of the average purity and efficiency cause the efficiency mainly depends on the etching depth. Although the etching depth is chosen to be 60 nm to make sure the optimum mode purity could be guaranteed in our experimental demonstration, to further improve the emitting efficiency of optical vortices based on such digitized nanostructure, increasing the etching depth reasonably would be the most effective way. It could be noticed that the maximum efficiency could reach 22% given the etching depth is 130 nm, just as shown in Figure 2(h) where the *y*-pol. OAM_+1_ mode is presented as an example. Another potential method to improve the emitting efficiency is adding a reflection layer to reduce the light field downward emitting [83]. Figure 2(h) also exhibits the simulation results when the scattering efficiency is compared with and without adding a thin gold film under the SiO_2_ substrate. Besides, there is no doubt that the emitting efficiency could also be improved by increasing the footprint of the designed nanostructure.

## 3 Results and discussion

**Figure 3:**
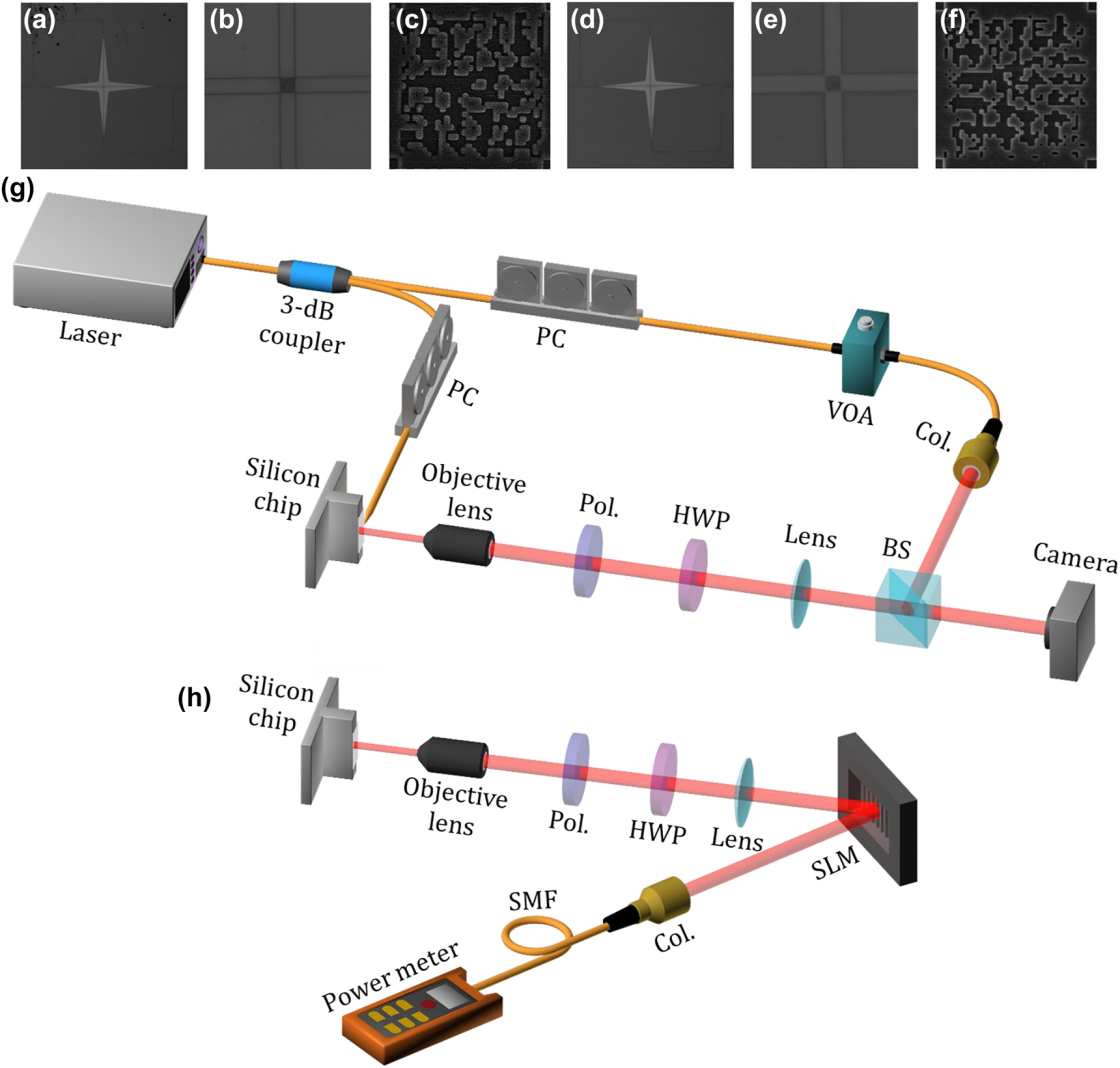
Fabricated devices and experimental setups for characterizing the generated wavelength-/polarization-/charge-diverse OAM modes. (a)–(c) Measured optical microscope images and scanning electron micrograph of the fabricated generator 1. (d)–(f) Measured optical microscope images and scanning electron micrograph of the fabricated generator 2. (g) Experimental configuration for measuring the intensity profiles and interferograms of the generated OAM modes. (h) Experimental configuration for measuring the mode crosstalk between the generated OAM modes. PC: polarization controller; VOA: variable optical attenuator; Col.: collimator; Pol.: polarizer; HWP: half-wave plate; BS: beam splitter; SLM: spatial light modulator; SMF: single-mode fiber.

Based on the optimized design of the digitized subwavelength surface structure proved by simulations, we fabricate the chip-scale optical vortex generator on an SOI wafer with a 220-nm-thick top silicon layer and a 2-μm-thick buried oxide layer. In the fabrication process, firstly the electron-beam-lithography (EBL) is employed to define the layout pattern and then an inductively coupled plasma (ICP) etching is followed to etch the top silicon layer. Waveguide outlines are etched fully down 220 nm to the buried oxide, while gratings are shallow etched down normally 60 nm. After that, a 1-μm-thick SiO_2_ layer is deposited on the top by plasma enhanced chemical vapor deposition (PECVD), covering the whole device as the upper cladding. Figure 3(a) and (b) depicts the measured microphotograph of the fabricated generator 1 for polarization diversity optical vortex generation, where the digitized subwavelength surface nanostructure region is connected by four adiabatic tapers from different directions for emitting specific OAM modes, respectively. The generator 2 for high-order optical vortex generation is fabricated with the similar digitized subwavelength surface structure, whose microphotograph is shown in Figure 3(d) and (e). Besides, the detailed scanning electron micrograph (SEM) images of the fabricated digitized subwavelength surface structures are shown in Figure 3(c) and (f), respectively.

Figure 3(g) and (h) shows the experimental setup for characterizing the fabricated wavelength-/polarization-/charge-diverse optical vortex generator, mainly used for testing the quality of generated OAM modes. Here two experimental configurations are utilized to comprehensively characterize the performance of the chip-scale optical vortex generator. In Figure 3(g), a tunable continuous-wave laser, enabling the broadband scanning of wavelength, is split into two branches by a 3 dB coupler. One branch is used to excite the in-plane guided fundamental TE_0_ mode in the waveguide with the input via the lensed fiber after a polarization controller (PC). With the edge coupling of light, OAM with specific polarization state and topological charge is coupled out and emitted into free space by the digitized subwavelength surface nanostructure. Considering the divergence of the surface emitted light, an objective lens is employed for collimation and another convex lens is used to adjust the beam size. Besides, a polarizer (Pol.) and a half-wave plate (HWP) are inserted into the light path so that the polarization state of the emitted optical vortex could be adjusted, while the other branch is coupled into free space by an optical fiber collimator (Col.) to provide a coherent reference Gaussian beam. This branch only consists of a variable optical attenuator (VOA) in order to appropriately adjust the power of the reference Gaussian beam to be comparable to the optical vortex. Then the interferogram could be captured by a camera after the two coherent light beams (optical vortex, reference Gaussian beam) are combined for interference with each other.

**Figure 4:**
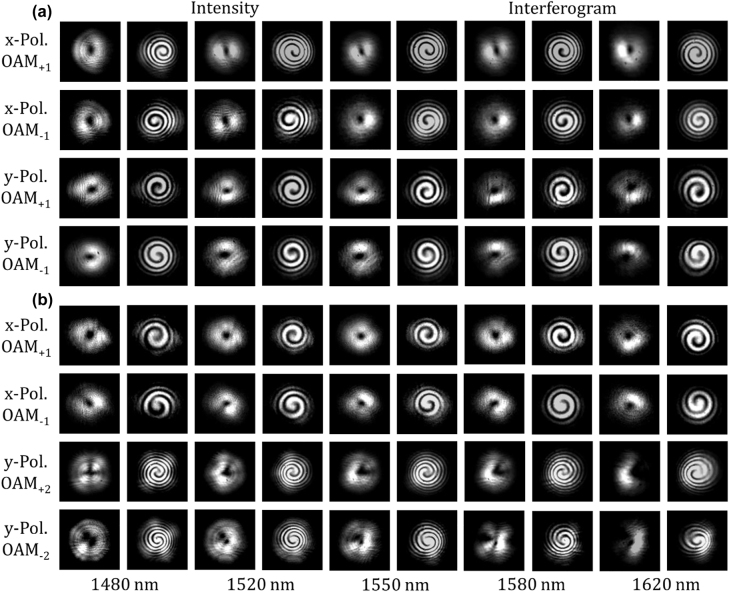
Measured far-field intensity profiles and interferograms of the generated OAM modes from 1480 to 1620 nm. (a) Measured results for the generated *x*-pol. OAM_+1_, *x*-pol. OAM_−1_, *y*-pol. OAM_+1_, and *y*-pol. OAM_−1_ mode, respectively. (b) Measured results for the generated *x*-pol. OAM_+1_, *x*-pol. OAM_−1_, *y*-pol. OAM_+2_, and *y*-pol. OAM_−2_ mode, respectively.

Shown in Figure 4(a) is the measured far-field intensity profiles and corresponding interferograms of the generated *x*-pol. OAM_+1_, *x*-pol. OAM_−1_, *y*-pol. OAM_+1_, and *y*-pol. OAM_−1_ mode ranging from 1480 to 1620 nm, respectively. Agreed with simulation results, the measured intensity profiles display a doughnut shape and the interferograms with spiral interference patterns indicate the helical phasefront of the generated OAM modes. It is noted that the sign of the topological charge is indicated by the chirality of the interference pattern. Besides, only slight degeneration of the mode quality is observed toward longer wavelength. Using the same experimental setup, we then experimentally study the wavelength-/polarization-/charge-diverse generation of high-order optical vortices by generator 2. The far-filed intensity profiles and interferograms are measured as shown in Figure 4(b). The first-order OAM modes (OAM_+1_, OAM_−1_) exhibit well-performed intensity and phase distributions in a wide wavelength range from 1480 to 1620 nm. On the other hand, the second-order OAM modes (OAM_+2_, OAM_−2_) show slight performance degradation in the intensity profiles, while the magnitude and sign of the OAM order can still be deduced from the measured interferograms within a wide wavelength range. It could be noticed that there exist some measured intensity profiles where a crescent shape is observed instead of a complete doughnut. This could be attributed to the objective lens employed for the collimation and focusing of the surface emitted light in the experimental setup, whose range of collecting emitted beam is limited to a certain extent. Besides, the emitting angle of optical vortex beams would make a difference as the working wavelength changes in a wide range from 1480 nm to 1630 nm, so that the edge part of the emitted optical vortex beam would be obscured when the working wavelength deviates from the central wavelength. To sum up, the measured intensity profiles and interferograms shown in Figure 4 confirm the successful generation of wavelength-/polarization-/charge-diverse optical vortices with favorable performance, i.e. OAM_+1_, OAM_−1_, OAM_+2_, and OAM_−2_ with two polarizations in a wide wavelength range (1480–1620 nm).

**Figure 5:**
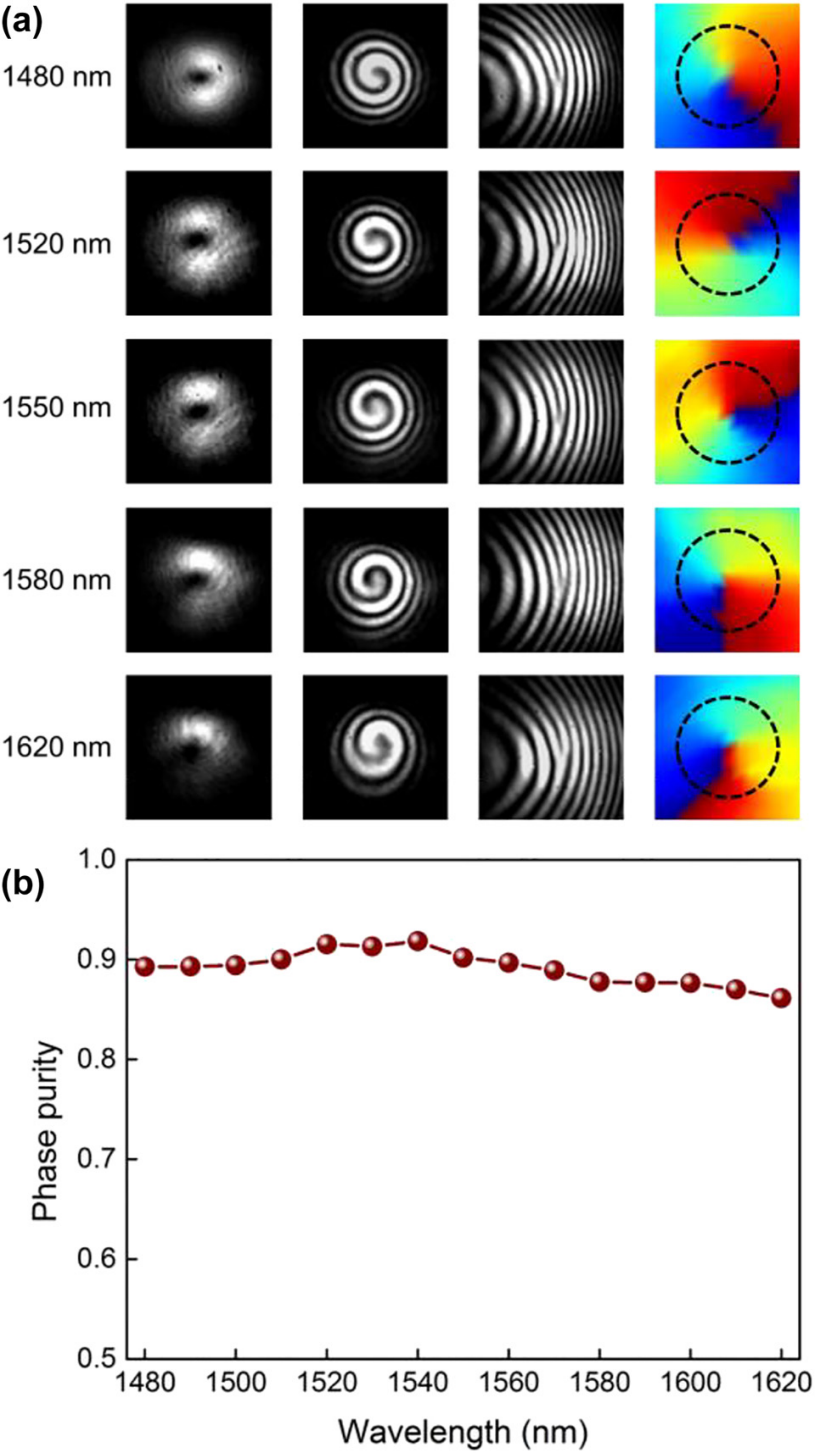
Reconstructed phase distribution and calculated phase purity of generated OAM modes. (a) Measured far-field intensity profiles, coaxial interferograms, tilt interferograms, and reconstructed phase distribution of the generated *y*-pol. OAM_−1_mode at 1480, 1520, 1550, 1580, and 1620 nm, respectively. (b) Measured phase purity of the generated *y*-pol. OAM_−1 mode_ versus wavelength.

Apart from the hollow shaped intensity profile with null value at the beam center, more attention should be focused on the phase purity of the OAM modes to evaluate the performance of our OAM generator. Although the spiral interference pattern makes its twisted spiral phase structure distinctly clear, here we characterize the phase purity numerically in a more precise way. Calculation of the phase purity is based on the extraction of its phase distribution, which could not be obtained directly. Here, we reconstruct the phase distribution from the fork interference fringe by a tilt interference with the Fourier transform method. As an example, results of the measurement with the generated y-pol. OAM_-1_ is shown in Figure 5. According to the monitored far-field intensity profiles shown in the first column, firstly the second column could be obtained easily by a strictly coaxial interference with a reference Gaussian light. Based on the test of spiral interference pattern, if the incident angle of either the two beams is adjusted to achieve a tilt interference, we can get the fork patterns shown in the third column. Then the phase information could be reconstructed with the Fourier transform method in the last column, whose azimuthal phase changes from 0 to 2*π* uniformly in a clockwise circle, indicting the favorable phase distribution of generated OAM_−1_ mode. Numerical calculation for the phase purity results in a maximum value of 0.92 at 1540 nm, no less than 0.86 ranging from 1480 to 1620 nm.

**Figure 6:**
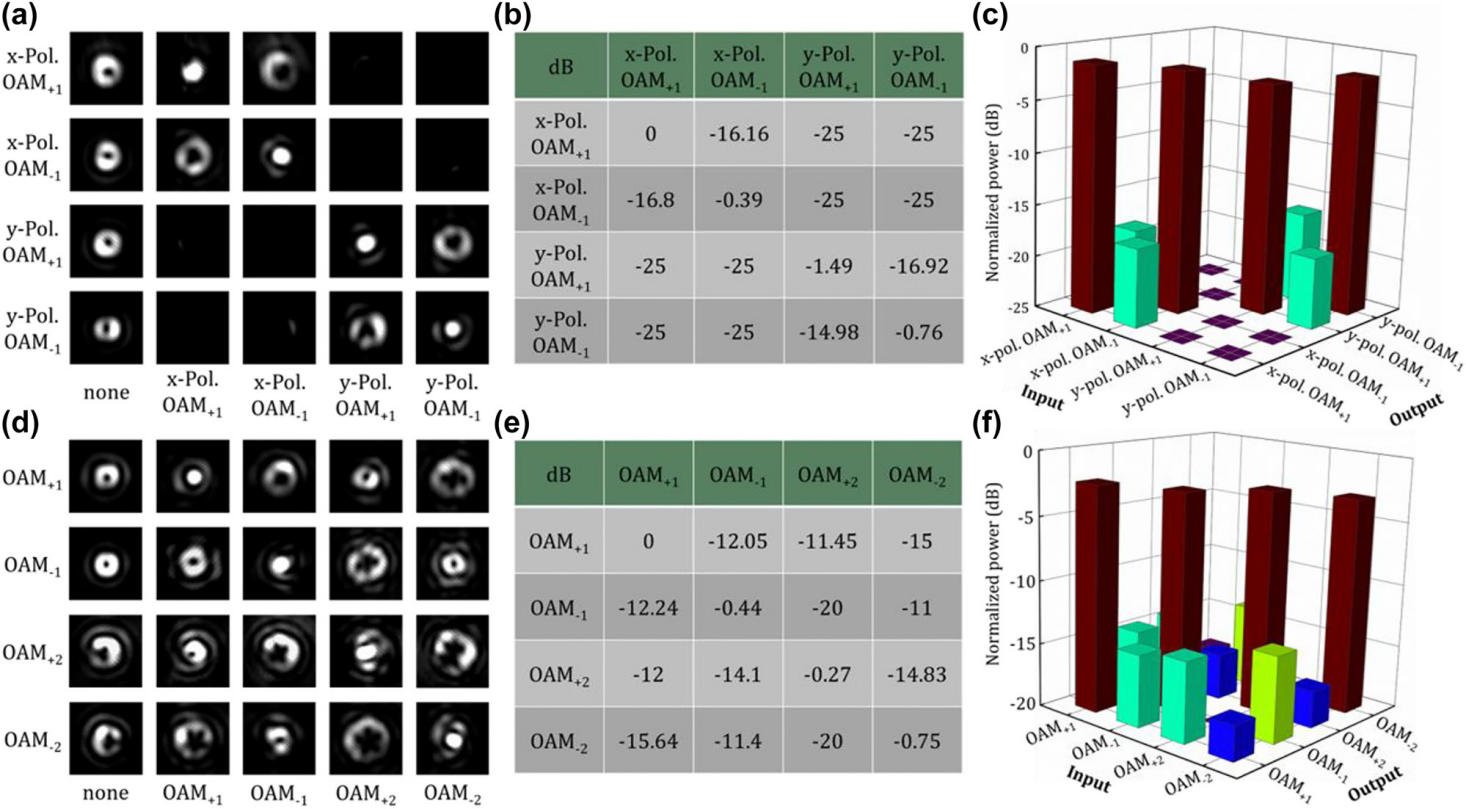
Experimental measurements of the mode crosstalk between the generated wavelength-/polarization-/charge-diverse OAM modes. (a)–(c) Measured results for the fabricated generator 1. (d)–(f) Measured results for the fabricated generator 2. (a) and (d) Measured intensity distribution matrix. (b) and (e) Measured 4 × 4 mode crosstalk matrix. (c) and (f) Measured histograms of the 4 × 4 mode crosstalk matrix.

The proposed chip-scale wavelength-/polarization-/charge-diverse optical vortex generator is expected to be used in multi-dimensional multiplexing optical communications, i.e. combined OAM multiplexing, WDM, and PDM. Hence, the crosstalk between different OAM modes is of great importance. Here, an experimental measurement method based on a spatial light modulator (SLM) is used to measure the mode crosstalk matrix. The experimental configuration is shown in Figure 3(f). The optical vortex emitted from the chip is demodulated by a polarizer (polarization demodulation) and an SLM loaded with a holographic phase pattern (OAM demodulation). Objective lens and convex lens are used to adjust the beam size projected onto the SLM. Note that the SLM is sensitive to polarization, so a polarizer and a half-wave plate are placed in the light path to adjust the light polarization to be aligned to the working direction of the SLM. The polarizer is set to be *x*- or *y*-direction for polarization demodulation and two kinds of pure phase patterns are selectively loaded onto the SLM to demodulate the OAM_+1_ or OAM_−1_ mode into Gaussian-like beam, respectively. Hence, as shown in Figure 6(a), the first column shows the far-field OAM intensity profiles without using a demodulation phase pattern. When a corresponding phase pattern is loaded onto the SLM, unlike OAM modes with doughnut shape intensity profile, the demodulated light after removing the helical phasefront displays a bright spot at the beam center, just like a Gaussian beam. The results with both a correct polarization demodulation and a correct OAM demodulation are shown in the diagonal elements (bright spots at the beam center) in Figure 6(a). By contrast, as shown in the off-diagonal elements in Figure 6(a), it is noted that the light beam remains a doughnut shape when using a noncorresponding demodulation phase pattern, and almost no light could be observed when setting the polarizer to an orthogonal direction. Hence, the mode crosstalk matrix measurement is followed by filtering and coupling only the central part of the demodulated beam into fiber connected with an optical power meter. According to the recorded mode crosstalk matrix in Figure 6(b) and (c), a crosstalk less than −14 dB is measured at the worst case. Similar measurement is carried out for the crosstalk between OAM_+1_, OAM_−1_, OAM_+2_, and OAM_−2_ modes generated by the high-order optical vortex generator, where holographic phase patterns for the demodulation of OAM_+1_, OAM_−1_, OAM_+2_, and OAM_−2_ modes are loaded onto the SLM instead. In the same way, light displays a bright spot at the beam center with a correct demodulation (diagonal), while a noncorresponding demodulation (off-diagonal) only results in null intensity at the beam center. The measured intensity distribution matrix is shown in Figure 6(d). The recorded mode crosstalk matrix is displayed in Figure 6(e) and (f). The crosstalk between any two OAM modes is less than −10 dB, and the lowest crosstalk is about −17 dB. The experimental results shown in Figures 4–6, especially the relatively low crosstalk between the wavelength-/polarization-/charge-diverse OAM modes, indicate the high-performance optical vortex generator and its great potential for multi-dimensional multiplexing optical communications (OAM multiplexing + WDM + PDM).

**Figure 7:**
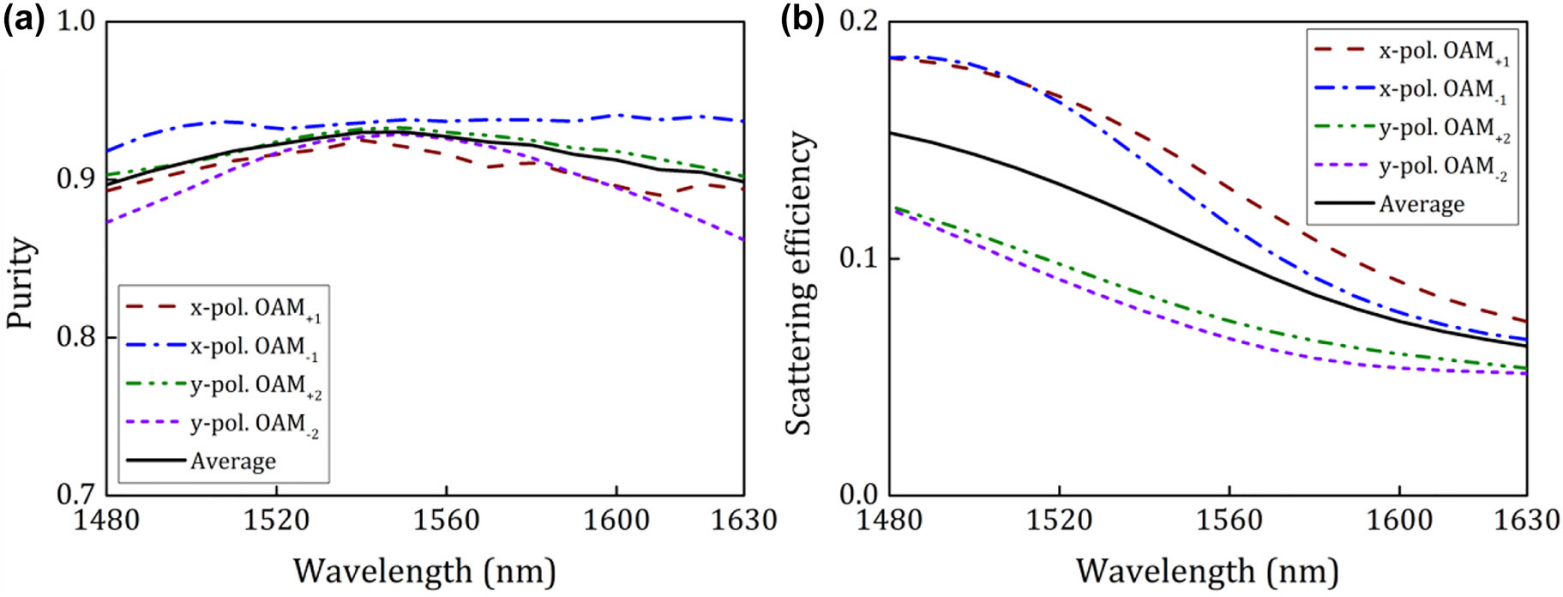
Simulation results of the generated *x*-pol. OAM_+1_, *x*-pol. OAM_−1_, *y*-pol. OAM_+2_, and *y*-pol. OAM_−2_ mode when the digitized subwavelength surface structure is optimized under the etching depth of 130 nm. (a) Calculated mode purity versus wavelength. (b) Calculated scattering efficiency versus wavelength.

**Figure 8:**
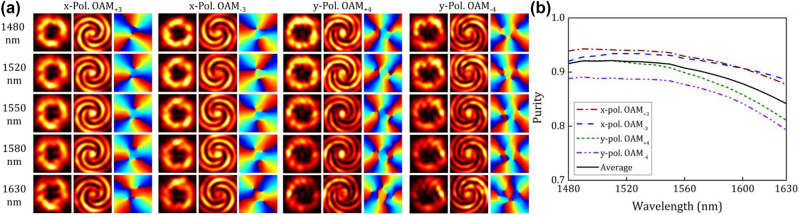
Simulation results for characterizing the wavelength-/polarization-/charge-diverse optical vortex generator of third-order and fourth-order OAM modes. (a) Intensity profiles, interferograms, and phase distributions of the generated *x*-pol. OAM_+3_, *x*-pol. OAM_−3_, *y*-pol. OAM_+4_, and *y*-pol. OAM_−4_ mode from 1480 to 1630 nm, respectively. (b) Calculated mode purity of the generated OAM modes versus wavelength.

The demonstrated wavelength-/polarization-/charge-diverse optical vortex generator based on digitized subwavelength surface structure shows outstanding performance. Some future perspectives toward a more robust optical vortex chip could be considered as follows:

(1) The nonlinear optimization algorithms might be further improved to design the silicon-based optical vortex generator more efficiently with superior performance in terms of mode purity and mode crosstalk. The scattering efficiency could also be improved by increasing the etching depth, just as shown in Figure 7 where the digitized subwavelength surface structure is optimized for the generation of *x*-pol. OAM_+1_, *x*-pol. OAM_−1_, *y*-pol. OAM_+2_, and *y*-pol. OAM_−2_ mode under the etching depth of 130 nm. It could be noticed that a favorable performance could also be achieved even when generating high-order OAM modes in the presence of the trade-off relationship between the efficiency and the purity. Besides, the scattering efficiency could be further improved by increasing the footprint of the designed nanostructure as well as by adding a reflection layer.
(2) Generation of much higher-order OAM modes could also be realized based on the proposed digitized subwavelength surface structure. As an example, the simulation results are given in Figure 8, where OAM modes with ±3 and ±4 topological charges are generated simultaneously. One can clearly see that although the mode purity degrades with the increase of wavelength, the average purity of the generated OAM modes is still larger than 0.92 at the central wavelength. Apart from much higher-order OAM modes, the basic concept of digitized subwavelength surface structure might be expanded to realize wavelength-/polarization-/charge-diverse optical vortex generator with even more input ports (>4) so that more OAM modes could be achievable using a single chip.
(3) High-efficient and low-crosstalk coupling and packaging of the fabricated optical vortex chip with fibers might be developed for practical applications, i.e. coupling and packaging of input ports with SMFs and vertical emission output with OAM fiber.
(4) The silicon-based optical vortex chip might be used both as the emitter and detector. In particular, assisted by the fiber-chip-fiber coupling and packaging, the proposed wavelength-/polarization-/charge-diverse optical vortex chip might be employed in practical multi-dimensional multiplexing fiber optical communications for capacity scaling.
(5) The digitized subwavelength surface structure on silicon platform might be further developed as a more generalized design method to realize much more complicated and robust functional photonic chip.

## 4 Conclusions

In summary, we propose and demonstrate CMOS-compatible ultra-compact integrated wavelength-/polarization-/charge-diverse optical vortex generator with a broadband wavelength range from 1480 to 1630 nm, supporting two polarizations and high-order OAM modes. Based on the designed digitized subwavelength surface structure optimized with the DBS algorithms, *x*-pol. OAM_+1_, *x*-pol. OAM_−1_, *y*-pol. OAM_+1_, and *y*-pol. OAM_−1_ mode could be generated with the mode purity exceeding 90%. Moreover, the generation of high-order OAM modes including OAM_+1_, OAM_−1_, OAM_+2_, and OAM_−2_ mode could be also realized with the same design method. We fabricate and comprehensively characterize the chip-scale wavelength-/polarization-/charge-diverse optical vortex generator. Outstanding performance is achieved in terms of mode purity and mode crosstalk. For the generation of *x*-pol. OAM_+1_, *x*-pol. OAM_−1_, *y*-pol. OAM_+1_, and *y*-pol. OAM_−1_ mode, a crosstalk less than −14 dB is obtained at the worst case. For the generation of OAM_+1_, OAM_−1_, OAM_+2_, and OAM_−2_ mode, the crosstalk between any two OAM modes is less than −10 dB, and the lowest crosstalk is about −17 dB. Moreover, we also show the possibility for the generation of much higher-order OAM modes (e.g. *x*-pol. OAM_+3_, *x*-pol. OAM_−3_, *y*-pol. OAM_+4_, and *y*-pol. OAM_−4_) using the digitized subwavelength surface structure. The demonstrated wavelength-/polarization-/charge-diverse optical vortex generator provides the possibility of fully accessing multiple physical dimensions of lightwaves such as wavelength, polarization, and space. The demonstrations open a way to implement multi-dimensional multiplexing optical communications with capacity scaling, i.e. incorporating compatible ultra-compact chip-enabled OAM multiplexing with well-established WDM and PDM techniques.
